# Clinical characteristics and risk factors analysis of 505 cases of infusion reactions in a tertiary hospital

**DOI:** 10.3389/fphar.2024.1292347

**Published:** 2024-02-06

**Authors:** Weiwei Yin, Bingqin Wen, Guoan Wang, Zhipeng Wang, Xuetao Kong, Yaozhou Wu, Xiao Meng, Xinyi Ou, Li Wei, Pengjiu Yu

**Affiliations:** ^1^ Department of Pharmacy, The First Affiliated Hospital, Guangzhou Medical University, Guangzhou, China; ^2^ School of Pharmaceutical Science, Guangzhou Medical University, Guangzhou, China; ^3^ National Clinical Research Center for Respiratory Disease, State Key Laboratory of Respiratory Disease, The First Affiliated Hospital, Guangzhou Institute of Respiratory Health, Guangzhou Medical University, Guangzhou, China

**Keywords:** infusion reactions, clinical characteristics, risk factors, pharmacovigilance, tertiary hospital

## Abstract

**Background:** The clinical characteristics and risk factors of infusion reactions (IRs) are inadequately described in clinical practice due to underreported cases. In the present study, we reported the current status of IRs based on an in-hospital pharmacovigilance database of a tertiary care hospital.

**Methods:** Our study conducted a retrospective analysis of drug-induced IRs recorded at an in-hospital pharmacovigilance center between January 2015 to December 2019. The descriptive statistical analysis encompassed main causative agents, clinical manifestations, organ/system involvement and outcome. The severity of IRs was assessed with reference to the CTCAE version 5.0 criteria and we investigated risk factors associated with severe IRs.

**Results:** During the study period, a total of 505 cases of inpatient drug-induced IRs were detected, of which 79.2% (400 cases) were classified as general IRs and 20.8% (105 cases) were categorized as severe IRs. The primary drugs responsible for these reactions were antibiotics (23%, 116 cases), with piperacillin sodium—sulbactam sodium being the most prevalent, followed by antineoplastic agents (18.4%, 93 cases) and traditional Chinese medicine injections (TCMIs) (12.9%, 65 cases). The administration of cefoperazone - sulbactam, mannatide, Shenqi Fuzheng, elemene, and diterpene ginkgolides meglumine resulted in a higher incidence of critical IRs. Among all cases of IRs, 43.2%, 41.2%, and 23.4% showed signs and symptoms of circulation, skin mucosa, and respiratory organs/systems, respectively. 9.1% of cases experienced systemic damage, while 7.1% and 5.9% of cases reported neurological and gastrointestinal related adverse reactions, respectively. The multivariate analysis revealed that alcohol consumption (OR = 2.389%, 95% CI 1.141–5.002, *p* = 0.021), age over 65 (OR = 1.814%, 95% CI 1.052–3.127, *p* = 0.032) and the utilization of contrast media (OR = 4.072%, 95% CI 1.903–8.713, *p* < 0.001) were identified as risk factors for the development of severe IRs.

**Conclusion:** Understanding the clinical characteristics of IRs helps to implement effective pharmaceutical monitoring and appropriate preventive measures for susceptible populations with risk factors.

## 1 Introduction

Intravenous infusion is a prevalent therapeutic modality in medical practice. Infusion reactions (IRs) encompass a spectrum of adverse events that occur during or following the administration of pharmacologically active substances or biologically active agents. These reactions may manifest either immediately during the infusion process or within a few hours to days post-administration. (Roselló et al., 2017). IRs were previously defined as unpredictable, also unrelated to dose and pharmacological activity of the drug, generally, they would be relieved or reversed when the treatment is terminated (Edwards and Aronson, 2000; Baldo, 2013). The two types of IRs can be classified as anaphylaxis, which is mediated by immunoglobulin E (IgE) antibody responses, and IgE non-dependent reactions (Joerger, 2012). Both types exhibit similar clinical symptoms that commonly affect the skin mucosa, respiratory system, circulatory system, gastrointestinal organs, and may pose life-threatening risks (Roselló et al., 2017).

A survey conducted by the Portuguese Pharmacovigilance System revealed that drug-induced anaphylaxis accounted for approximately 6% of all adverse drug reactions (ADRs), with antibiotics being reported as the most common causative agents, followed by acetaminophen and antineoplastics. Furthermore, contrast medium emerged as a significant contributor to allergic events (Ribeiro-Vaz et al., 2013). According to the latest research from the US Food and Drug Administration Adverse Event Reporting System, major agents associated with anaphylaxis include antibiotics, monoclonal antibodies, and non-steroidal anti-inflammatory drugs, while antibiotics, radiocontrast agents, and intraoperative drugs were linked to fatal allergic reactions (Yu et al., 2021). Additionally, the field of allergic reactions focuses on recognizing and exploring risk factors. Numerous studies have demonstrated a significant association between advanced age, coexisting asthma, and underlying cardiovascular disease with the occurrence of severe or fatal anaphylaxis (Turner et al., 2017; Kim et al., 2018; Worm et al., 2018; Pflipsen and Vega Colon, 2020). The evaluation of an in-hospital pharmacovigilance database from a tertiary care hospital in Korea shown that the use of iodine-containing contrast agents and neuromuscular blocking agents were regarded as potential risk factors for the development of anaphylactic shock (Park et al., 2017). Given that allergic reactions are a subset of IRs, it is plausible to postulate the presence of similar drug triggers and risk factors in IRs, thereby necessitating an exploration for reliable causative drugs and risk factors.

Successive reports have documented the occurrence of pegloticase, vancomycin, and intravenous iron-related IRs (Baraf et al., 2014; Alvarez-Arango et al., 2021; Stojanovic et al., 2021). The clinical features description and risk causes cognition of IRs, however, have received limited attention. The objective of this retrospective analysis of real-world data is to provide a comprehensive report on the current status of IRs, including the main culprit agents, clinical symptoms, organ/system involvement, outcome and regression. Additionally, it aims to evaluate the risk factors related to severe IRs. Understanding the clinical characteristics of IRs can help with active drug monitoring and adequate preventive measures for vulnerable groups with risk factors.

## 2 Methods

### 2.1 Clinical data

**FIGURE 1:**
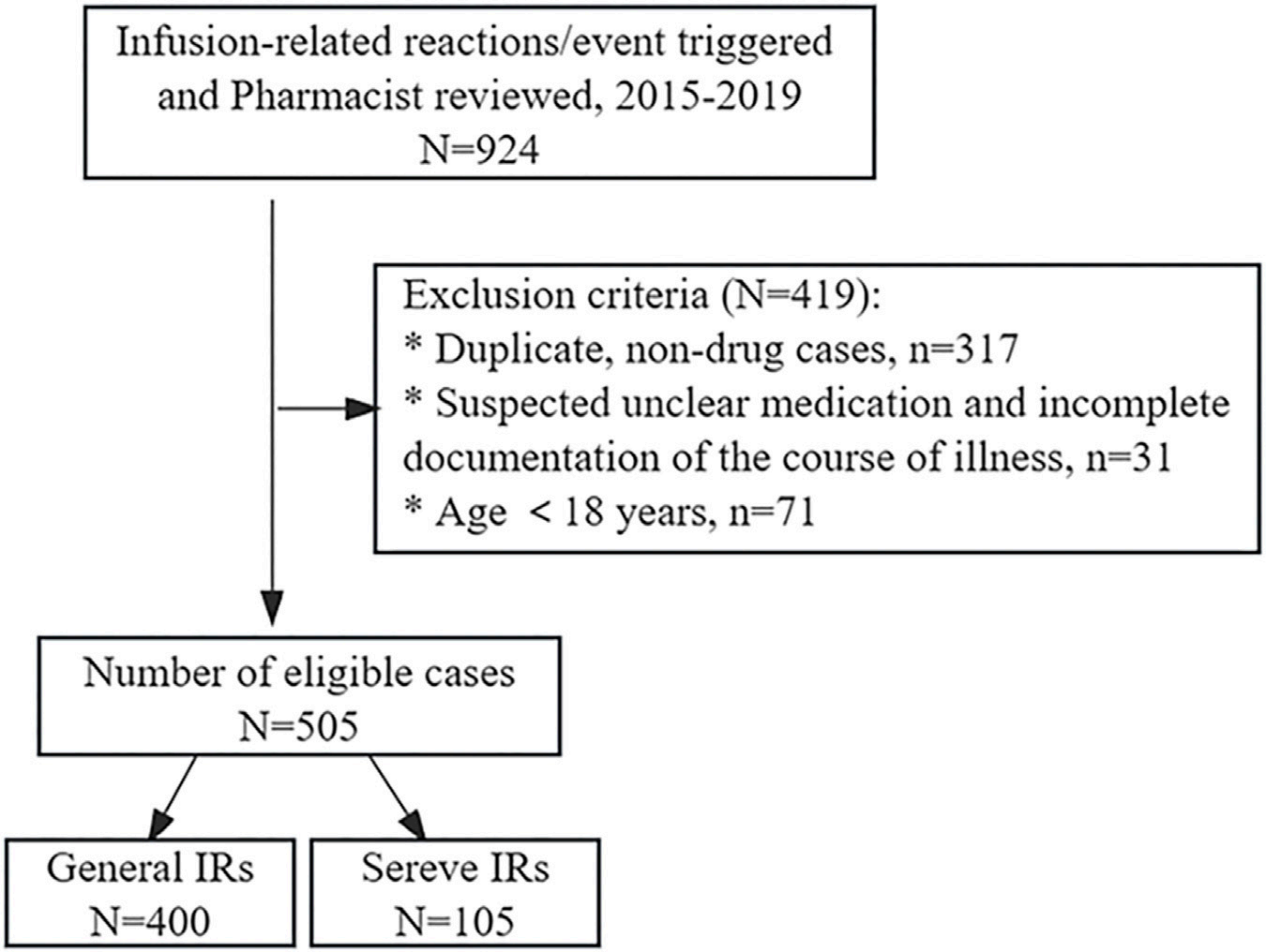
Flowchart of case selection.

This retrospective study was based on the in-hospital pharmacovigilance system, setting triggers for infusion-related reactions/events triggers, and spanned the period from January 2015 to December 2019, with a total of 924 cases reviewed by pharmacists eligible for inclusion. Repeat reports and non-drug-induced adverse reactions, such as events related to blood transfusions and transfusion ports, were excluded. In addition, cases aged <18 years, suspected unknown drugs, and incomplete disease course were excluded (Figure 1). The patient’s demographic and clinical data, including age, gender, history of drug-related allergies, smoking status, alcohol consumption history, co-morbidities, initial suspected medication, anti-allergic pretreatment regimen, eosinophil count, clinical presentations, outcome and regression of IRs were systematically recorded. The study was reviewed and approved by the First Affiliated Hospital of Guangzhou Medical University ethics committee (No. ES-2023-146-01).

### 2.2 Assessment of IRs

The assessment was conducted in accordance with the National Cancer Institute common terminology criteria for adverse events (CTCAE, version 5.0). Our study protocol defines the classification of IRs as follows: Grade 1 refers to transient and mild reactions that do not necessitate interruption of the infusion, such as reducing the infusion dose or slowing down the infusion rate, and do not require any specific treatment. Grade 2 requires therapy or suspension of infusion, but can be rapidly alleviated with symptomatic treatment (e.g., antihistamines, glucocorticoids, intravenous fluids, supportive care) and prophylactic medication for less than 24 h. Grade 3 represents serious or medically important, yet not immediately life-threatening, characterized by prolonged symptom duration, poor response to symptomatic treatment, and/or relapse after initial improvement. Grade 4 indicates life-threatening and deserving urgent treatment. Grade 5 denotes death connected with an adverse event. Of these, grades 1 and 2 are categorized as general IRs, while grades 3, 4, and 5 are recognized as severe IRs.

### 2.3 Statistical analysis

The count data were statistically described by frequency and compared using Pearson’s chi-square test and Fisher’s exact test for univariate analysis. Variables with *p*-values <0.2 were screened, and logistic regression analysis was performed to determine risk factors relevant to severe IRs, with severity of IRs as the dependent variable. One-way ANOVA was employed to compare the difference in eosinophil count between groups. Statistical analyses were performed using SPSS 26.0 software (IBM, Armonk, NY, USA, RRID: SCR_016479), considering *p*-values <0.05 as statistically significant.

## 3 Results

### 3.1 Demographic and clinical characteristics of drug-induced IRs

A total of 505 cases of IRs were retrieved. The severity of IRs was evaluated based on CTCAE version 5.0, of which 400 cases (79.2%) classified as mild and 105 cases (20.8%) categorized as severe. Of these patients, 151 cases (29.9%) were aged between 18 and 50 years, while 188 cases (37.2%) fell within the age range of 50–65 years. Additionally, 166 patients (32.9%) were aged over 65. Adverse reactions resulting from intravenous drug infusion were predominantly observed in patients aged 50 and above. The gender composition of male and female patients was similar, with a history of drug-induced allergies reported in 110 cases (21.8%). The majority of combined diseases were hypertensive in nature. Anti-allergic premedication was administered to 106 cases (21%), comprising 92 cases of general IRs and 14 cases of severe IRs (Table 1).

**TABLE 1:** Demographic and clinical characteristics.

	Total number N = 505	IRs		P-value^ξ^
		General	Severe	
		N = 400 (79.2)	N = 105 (20.8)	
Age(y)				0.075
18≤age≤50	151 (29.9)	123 (30.8)	28 (26.7)	
50<age≤65	188 (37.2)	139 (34.8)	49 (46.7)	
>65	166 (32.9)	138 (34.5)	28 (26.7)	
Sex				0.408
Male	256 (50.7)	199 (49.8)	57 (54.3)	
Female	249 (49.3)	201 (50.3)	48 (45.7)	
History of drug-derived allergy				0.273
Have	110 (21.8)	83 (20.8)	27 (25.7)	
None	395 (78.2)	317 (79.3)	78 (74.3)	
Smoking status				0.426
Have	143 (28.3)	110 (27.5)	33 (31.4)	
None	362 (71.7)	290 (72.5)	72 (68.6)	
History of drinking				0.008
Have	37 (7.3)	23 (5.8)	14 (13.3)	
None	468 (92.7)	377 (94.3)	91 (86.7)	
Pretreatment medication				0.03
Have	106 (21)	92 (23)	14 (13.3)	
None	399 (79)	308 (77)	91 (86.7)	
Co-morbidities				
Airway diseases^δ^	56 (11.1)	45 (11.3)	11 (10.5)	0.822
Ischemic heart disease	81 (16)	60 (15)	21 (20)	0.214
Hypertension	148 (29.3)	116 (29)	32 (30.5)	0.767
Diabetes	61 (12.1)	50 (12.5)	11 ((10.5))	0.571
Culprit drugs				
Antibiotics	149 (29.5)	128 (32)	21 (20)	0.16
Antineoplastics	93 (18.4)	73 (18.3)	20 (19)	0.851
Contrast medium	35 (6.9)	17 (4.3)	18 (17.1)	<0.001
Nutrients	22 (4.4)	19 (4.8)	3 (2.9)	0.564
Biological preparations	36 (7.1)	30 (7.5)	6 (5.7)	0.527
TCMIs	65 (12.9)	46 (11.5)	19 (18.1)	0.072

δ, Asthma or Chronic obstructive pulmonary disease (COPD). ξ, Pearson’s chi-square test and Fisher’s exact test. TCMIs, Traditional Chinese Medicine Injections.

### 3.2 The main causative agents of IRs

Antibiotics (116/23%) were the primary causative agents for in-hospital IRs. Among the antibiotics, piperacillin sodium-sulbactam sodium was the most frequently implicated, while cefoperazone sodium-sulbactam sodium exhibited the highest propensity for severe IRs. The second most often reported antineoplastic agents (93/18.4%) were predominantly characterized by the high prevalence of platinum compounds and mannatide, with mannatide displaying the highest number of instances resulting in severe IRs. Subsequently, Traditional Chinese Medicine Injections (TCMIs) were observed in 65 cases (12.9%), of which Sulfotanshinone sodium was the principal trigger for IRs. However, severe IRs were mainly caused by Shenqi Fuzheng, elemene and diterpene ginkgolides meglumine. Furthermore, more than half of the severe IRs were attributed to intravenous infusion of contrast media. The main causative agents of IRs may be associated with the overall frequency of utilization. However, there does not appear to exist a correlation between the frequency of utilization and the severity of IRs (Table 2).

**TABLE 2:** Details of the distribution of the main causative drugs.

Drug name	Total patients of drug use (N)	Number of IRs (with severe)
Antibiotics		149 (21)
Piperacillin-Sulbactam/Tazobactam	29876	46 (6)
Cefoperazone-Sulbactam	14266	15 (5)
Cefmetazole	18005	9 (1)
Cefuroxime	21972	7 (2)
Ceftazidime	3254	5 (0)
Cefazolin	22536	5 (0)
Vancomycin	3584	5 (0)
Cefathiamidine	22944	4 (1)
Meropenem	13199	4 (1)
Teicoplanin	2079	4 (0)
Imipenem And Cilastatin	6630	3 (0)
Azithromycin	3507	3 (0)
Latamoxef	9574	2 (1)
Clindamycin	4268	2 (0)
Cefepime	766	1 (0)
Aztreonam	-	1 (0)
Levofloxacin	40740	16 (2)
Moxifloxacin	19978	14 (1)
Ciprofloxacin	2629	3 (1)
Antineoplastic agents		93 (20)
Platinum compounds	39114	32 (4)
Mannatide	16387	25 (8)
Monoclonal antibodies	2124	14 (4)
Paclitaxel	8086	8 (1)
Doxorubicin	89	5 (1)
Docetaxel	7256	2 (0)
Recombinant Human Tumor Necrosis Factor	3686	2 (1)
Recombinant Human Interleukin-2 (125Ala)	615	1 (0)
Group A *Streptococcus*	1027	1 (0)
Etoposide	5307	1 (0)
Thymalfasin	11098	1 (0)
Cytarabine	782	1 (1)
TCMIs		65 (19)
Sulfotanshinone Sodium	30174	13 (1)
Shenqi Fuzheng	21387	9 (4)
Cervus and Cucumis Polypeptide	10257	6 (3)
Aidi	8005	6 (1)
Elemene	3182	5 (3)
Compound Kushen	6048	5 (0)
Diterpene Ginkgolides Meglumine	2239	3 (2)
Lentinan	14219	3 (1)
Shuxuetong	3882	3 (1)
Xueshuantong	8523	3 (1)
Kangai	8552	3 (1)
Xiyanping	3824	2 (1)
Canfu	1987	2 (0)
Kanglaite	10066	1 (0)
Xingnaojing	7429	1 (0)
Contrast media	4705	35 (18)

Note: Our study counted only the top four major causative drugs.

### 3.3 Organ/system involvement and major clinical signs of IRs

Among the 505 cases included in this retrospective analysis, 43.2%, 41.2%, and 23.4% experienced signs and symptoms of circulatory, cutaneous mucosal, and respiratory, respectively. In addition, systemic damage was reported in 9.1% of cases. While neurological and gastrointestinal adverse reactions occurred in 7.1% and 5.9% of cases, respectively. Detailed clinical manifestations are provided in Table 3.

**TABLE 3:** Involving organs/systems and the main clinical manifestations.

Involved organs/systems	Clinical symptoms	Number (percentage)
Skin mucosa system	Rash, itching, transient skin flushing, congestion and swelling of the mucous membranes of the nose/eyes/throat	208 (41.2)
Digestive system	Nausea, vomiting, abdominal pain	30 (5.9)
Respiratory system	Chest tightness, shortness of breath, difficulty breathing, cough	118 (23.4)
Circulatory system	Chills, fever, sweating, decreased oxygen/blood pressure, tachycardia, cyanosis, syncope, pallor	218 (43.2)
Nerves system	Dizziness, headache, weakness, convulsions, confusion or restlessness, incontinence	36 (7.1)
Systemic damage	Anaphylactic shock	46 (9.1)

Note: The total number of cases of system-organ involvement is greater than the total number of ADR, reports because more than one type of system-organ damage may occur in a single ADR, report.

**FIGURE 2:**
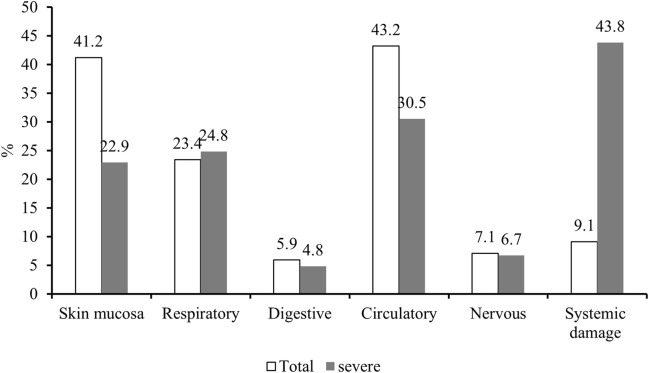
Organ/system involvement in IRs.

The details of both overall and severe IRs involving various organ systems are presented in Figure 2, revealing a notable predominance of severe IRs affecting the respiratory system and significantly heightened systemic damage compared to the overall incidence of IRs.

**FIGURE 3:**
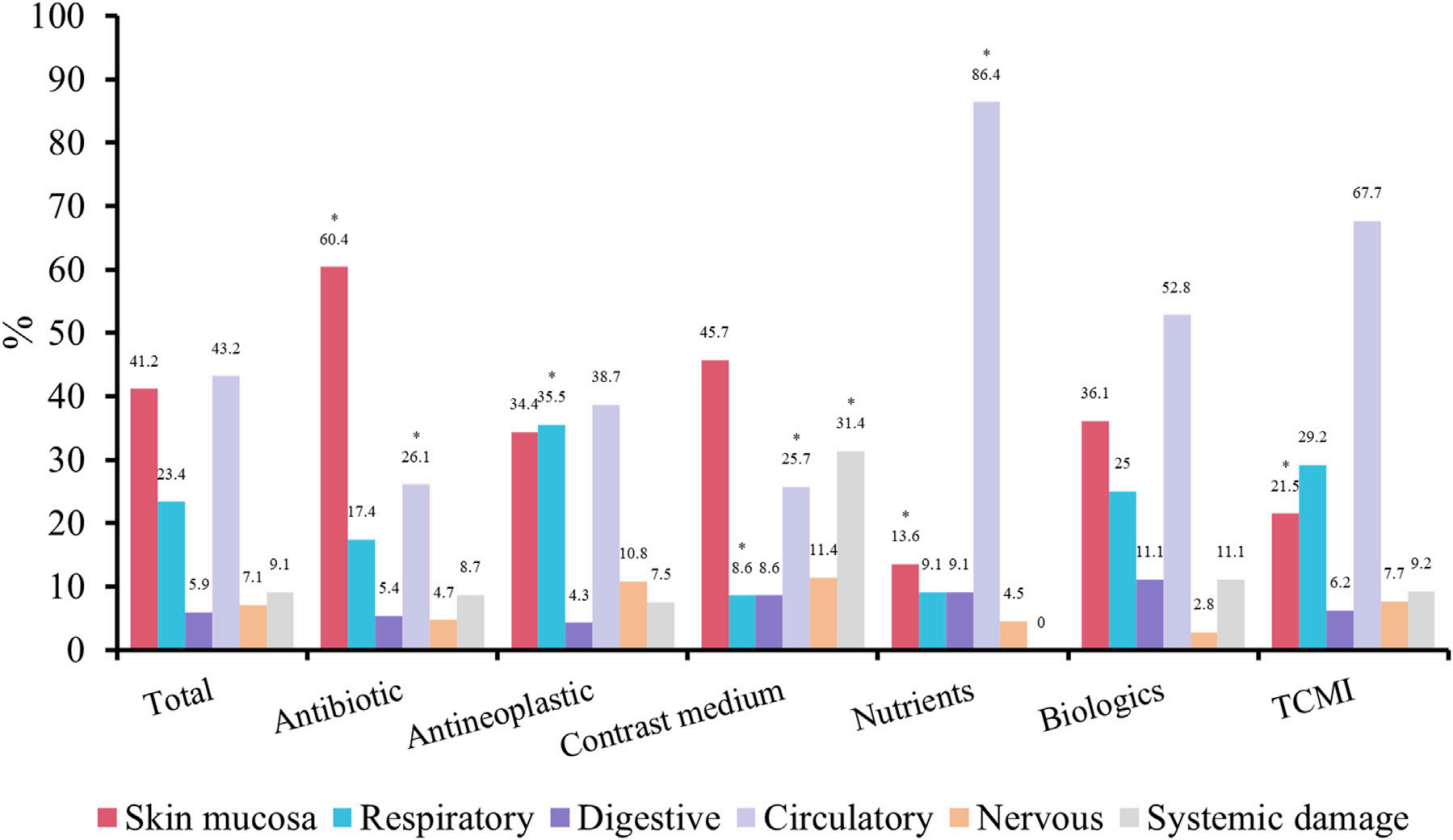
Symptoms and signs of IRs. *p < 0.05 compare with total patients. TCMI, Traditional Chinese medicine injection.

The administration of antibiotics was predominantly associated with skin and mucosal adverse reactions, while being less correlated with circulatory events. Conversely, the use of antineoplastic agents primarily exhibited relevance to respiratory events. Moreover, the application of contrast agents often accompanied by systemic damage and less related to respiratory and circulatory events. In contrast, the side effects linked to the administration of nutritional drugs and herbal injections showed the opposite pattern to that of antibiotics (Figure 3).

### 3.4 Outcome and regression

General IRs are typically transient and mild, and do not necessitate treatment discontinuation or complete resolution of signs and symptoms in all cases following interruption of infusion or management. Conversely, severe IRs often manifest as life-threatening events subsequent to intravenous drug administration, demanding immediate therapeutic intervention. Most of these cases were resolved through a series of clinical pathway interventions, including discontinuation of the suspected drug infusion, administration of intravenous fluids, anti-allergy medications (such as corticosteroids/antihistamines), and adjunctive support. However, a minority of cases exhibited unsatisfactory management outcomes with prolonged symptom duration and even two adverse event-related deaths (Table 4).

**TABLE 4:** Outcome and regression.

Ending transitions	IRs	
	General	Severe
	N = 400	N = 105
Improvement	400 (100)	74 (70.5)
Sustained	0	29 (27.6)
Death	0	2 (1.90)

### 3.5 Risk factors for severe IRs

A total of 105 cases of severe IRs were reported. There was a significant association between intravenous administration of contrast media and an elevated risk of systemic damage. In addition, the administration of antineoplastic agents was found to induce severe IRs specifically affecting the respiratory system. Among these cases, cefoperazone-sulbactam, mannatide, Shenqi Fuzheng, elemene, and diterpene ginkgolides meglumine were relatively more serious IRs triggered by antibiotics, antineoplastic drugs, and TCMIs, respectively.

The factors influencing severe IRs were further investigated through logistic regression analysis, aiming to identify the risk factors associated with serious IRs. Univariate analysis was conducted to screen for variables with a significance level of *p* < 0.2, including age, alcohol consumption, use of antibiotics, contrast agents and TCMIs, as well as whether or not premedication was administered. Multivariate analysis confirmed that alcohol consumption (OR = 2.389%, 95% CI 1.141–5.002, *p* = 0.021), age over 65 (OR = 1.814%, 95% CI 1.052–3.127, *p* = 0.032) and contrast media use (OR = 4.072%, 95% CI 1.903–8.713, *p* < 0.001) were substantial risk factors associated with the development of serious IRs (Table 5).

**TABLE 5:** Multifactorial logistic regression analysis of severe IRs.

Covariate	B	S.E.	Wald	OR	95% CI		*p*-value
					Lower	Upper	
Age							
18≤age≤50				Reference			
50<age≤65	0.190	0.307	0.382	1.209	0.662	2.206	0.537
>65	0.595	0.278	4.586	1.814	1.052	3.127	0.032
Drink	0.871	0.377	5.337	2.389	1.141	5.002	0.021
Antibiotic	−0.320	0.298	1.156	0.726	0.405	1.302	0.282
Contrast medium	1.404	0.388	13.088	4.072	1.903	8.713	<0.001
TCMI	0.433	0.329	1.732	1.543	0.809	2.942	0.188
Pretreatment	−0.591	0.333	3.150	0.554	0.289	1.064	0.076

Pretreatment refers to the application of antineoplastic drugs for prophylaxis, such as antihistamines, glucocorticoids, anti-inflammatory drugs.

### 3.6 Changes in eosinophil count

**FIGURE 4:**
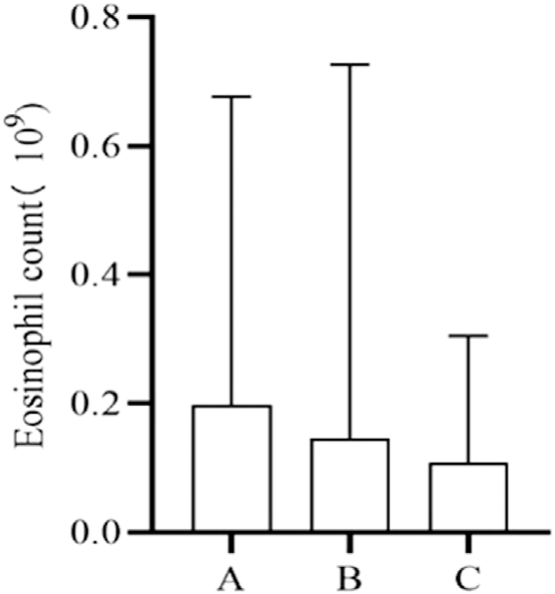
Changes in eosinophil count. A, Baseline level before IRs. B, Time level of IRs. C, Level after IRs.

The number of cases that fulfilled the criteria for testing serum eosinophil counts at, before, and after the onset of the IR was 106. Multiple measurements were averaged to compare the differences between these three time points. However, no statistically significant differences were observed (Figure 4).

## 4 Discussion

Our study retrospectively reported the current status of IRs based on domestic real-world data from hospital pharmacovigilance centre. Studies derived from domestic and foreign pharmacovigilance databases have consistently found the primary causative agents responsible for allergic reactions, which align with the characteristics of drugs that triggered IRs in this investigation (Ribeiro-Vaz et al., 2013; Wang et al., 2017). A majority of previous studies have demonstrated that β-lactam antibiotics were the culprit drugs of anaphylaxis (Renaudin et al., 2013; Ribeiro-Vaz et al., 2013; Turner et al., 2017), and one study suggested that piperacillin, a penicillin derivative, was the most frequently reported drug (Park et al., 2021). The current study indicated that antibiotics were the most common causative agents of IRs, with piperacillin sodium-sulbactam sodium being recorded in the greatest frequency, revealing parallels between the profiles of the drugs triggering IRs and the prevalent drugs formerly described as allergic reactions. Therefore, clinical use of medications should be alert to and prevent the possibility of IRs induced by intravenous infusion of allergy-prone drugs.

The idea that the skin mucosa is the organ/system most significantly affected by ADRs to antibiotics is consistently supported by a large number of studies (Diaz and Ciurea, 2012; Zhao et al., 2019; Jourdan et al., 2020). Therefore, meticulous attention was given to possible skin and mucosal side effects such as rash, pruritus, and flushing during intravenous infusion. Meanwhile, piperacillin, levofloxacin, cefoperazone and moxifloxacin were reported mostly antibiotics in present study. Notably, cefoperazone was the culprit of severe IRs, which need to be strengthened to prevent and monitor the drugs that cause frequent and serious ADRs.

Although platinum compounds were the primary cause of IRs among antineoplastic agents, mannatide induced the highest number of severe IRs. This may be attributed to adherence to clinical dosing principles or institutional policies, where routine administration of classical antineoplastic agents such as platinum compounds, paclitaxel, docetaxel, and monoclonal antibodies significantly mitigated the risk of IRs associated with chemotherapy drugs. The available evidence suggests that a combination of prophylactic strategies, including antihistamines, H2 antagonists, leukotriene receptor antagonists, corticosteroids, and other reasonable interventions, can effectively reduce the incidence and severity of chemotherapy drug-induced IRs while enhancing safety (ALMuhizi et al., 2022). Mannatide, a glycopeptide compound distinct from conventional anti-tumour drugs. A study on the pro-inflammatory response and chemotaxis of mannatide demonstrated its potential impact on the severity of IgE-mediated diseases, including allergic reactions (Żelechowska et al., 2021). The findings of this study caution that mannatide was susceptible to severe IRs, and this product has been linked to ADRs such as hypersensitivity responses, chest tightness and dyspnea. Therefore, it is recommended to use under close physician supervision and with resuscitative measures.

TCMIs are an extension and development of traditional Chinese medicine, boasting a rich history spanning over 70 years. As of 2017, the sale of TCMI products has been authorized for a total of 134 generic names from 224 reputable manufacturers (Li et al., 2018). The utilization of TCMIs, however, has resulted in a growing number of documented IRs (Li et al., 2019). The results of this study demonstrated that TCMIs are important triggers of IRs, often involving the circulatory system. This suggests that TCMIs are becoming more prevalent in clinical use, however, continued attention to ADRs is warranted, common clinical manifestations include chills, fever, sweating, reduced blood oxygen levels/pressure, tachycardia, cyanosis, syncope and pallor.

According to a study conducted between 2014 and 2019 on the safety of TCMIs, Shenmai, Xiangdan, Danshen, Shengmai, Huangqi, and Xuebijing injection exhibited a higher proportion of severe ADRs compared to the average (Huang et al., 2021). The findings are inconsistent with current research, which reported a higher incidence of ADRs associated with Shenqi Fuzheng, Elemene, and diterpene ginkgolides meglumine injection. The variation in culprit drugs was inferred to reflect the diversity in in-hospital drug utilization patterns among tertiary medical centers. Furthermore, there are discrepancies in the drug characteristics summary between individual hospitals and provincial municipalities. Therefore, it is imperative to implement targeted drug monitoring during the clinical application of Shenqi Fuzheng, elemene, and diterpene ginkgolides meglumine herbal injections to mitigate the occurrence of severe IRs.

Previous studies have primarily focused on the risk factors of individual drug-induced IRs, such as infliximab (Duron et al., 2015; Wang et al., 2019) and cetuximab (Touma et al., 2014). The present study investigated the risk factors of severe IRs, and established alcohol consumption, age over 65 and the application of contrast media as risk factors for severe IRs. The reports stated that drinking may interfere with the absorption, distribution, metabolism and excretion of drugs, thereby increase the likelihood of ADR (Weathermon and Crabb, 1999; Castle et al., 2016). To prevent alcohol-drug interactions, it is recommended to avoid consuming alcohol while taking drugs that interact with it. Drug use varies by age. Polypharmacy is very common in the elderly, which may contribute to mainly the growth in the severity of IRs in older individuals (Jerschow et al., 2014). Among pathogenic medications, the use of contrast agents was found to increase the vulnerability to critical IRs, which may be attributed to their inherent properties. The *p*-value for univariate analysis of anti-allergic pretreatment was 0.03, while for multivariate analysis it approached 0.05, suggesting a potential protective effect against drug-induced severe IRs. However, due to limitations in sample size, this difference was not statistically significant. Moreover, several studies have identified comorbidities such as asthma, chronic obstructive pulmonary disease (COPD), and cardiovascular disorders as significant risk factors for severe allergic reactions (Simons et al., 2011; Turner et al., 2017). In this study, airway disease (asthma/COPD), hypertension, diabetes mellitus, and ischemic heart disease were assessed as co-morbidities increasing the risk of severe IRs, however, these differences did not reach statistical significance. Adverse event-related deaths occurred in 2 cases, mainly owning to patients suffering from an underlying disease that did not progress optimistically progressing to cardiac arrest, with the corresponding medication considered as a secondary factor.

Serum eosinophilia can arise from various drug reactions, including but not limited to NSAIDs, antibiotics, and anticonvulsants (Gottlieb et al., 2022; Hama et al., 2022; Awad et al., 2023). We endeavored to assess alterations in eosinophil counts following drug-induced IRs, unfortunately, no statistically significant differences were observed when comparing eosinophil counts before, during, and after the onset of IRs. This analysis may be attributed to the fact that the causative drugs of IRs are not among the primary agents known to induce serum eosinophilia.

This study has several limitations. Firstly, it was a retrospective analysis, which inevitably introduces bias and confounding factors. Additionally, accurately tracking the time interval between drug infusion and onset of adverse reactions posed challenges. Moreover, the rate of infusion may be considered an influential factor in IRs. To overcome these constraints and provide a more precise evaluation of severe IR risk factors, large-scale prospective studies are warranted.

In conclusion, the present study retrospectively reported an update on IRs based on domestic real-world data from hospital pharmacovigilance center and demonstrated antibiotics, antineoplastic agents and TCMIs as the prime culprit drugs in the tertiary care center, with a relatively high number of drugs triggering serious IRs including cefoperazone and sulbactam, mannatide, Shenqi Fuzheng, elemene and diterpene ginkgolides meglumine. The occurrence of IRs may be associated with the total number of medications administered. However, no such correlation seems to exist in terms of severity. In addition, alcohol consumption, age over 65 and the use of contrast media were risk factors of serious IRs. Therefore, reaching a comprehensive understanding of the clinical characteristics of IRs will facilitate active pharmacovigilance and the implementation of appropriate preventive measures for susceptible groups with risk factors.

## Data Availability

The raw data supporting the conclusion of this article will be made available by the authors, without undue reservation.
